# Post-adoption experiences of discrimination moderated by sleep quality are associated with depressive symptoms in previously institutionalized youth over and above deprivation-induced depression risk

**DOI:** 10.1017/S0954579424000932

**Published:** 2024-06-04

**Authors:** Mirinda M. Morency, Bonny Donzella, Brie M. Reid, Richard M. Lee, Donald R. Dengel, Megan R. Gunnar

**Affiliations:** 1 Institute of Child Development, University of Minnesota Twin-Cities, Minneapolis, MN, USA; 2 Department of Psychiatry and Human Behavior, Warren Alpert Medical School, Brown University, Providence, RI, USA; 3 Department of Psychology, University of Minnesota Twin-Cities, Minneapolis, MN, USA; 4 Center for Pediatric Obesity Medicine, Minneapolis, MN, USA; 5 Department of Pediatrics, University of Minnesota Medical School, University of Minnesota Twin-Cities, Minneapolis, MN, USA

**Keywords:** autonomic balance, adolescents, discrimination, depression, early life stress, institutional rearing, sleep, transracial adoption

## Abstract

The association of post-adoption experiences of discrimination with depressive symptoms was examined in 93 previously institutionalized (PI) youth (84% transracially adopted). Additionally, we explored whether sleep quality statistically moderated this association. Notably, we examined these associations after covarying a measure of autonomic balance (high/low frequency ratio in heart rate variability) affected by early institutional deprivation and a known risk factor for depression. PI youth exhibited more depressive symptoms and experiences of discrimination than 95 comparison youth (non-adopted, NA) raised in their biological families in the United States. In the final regression model, there was a significant interaction between sleep quality and discrimination, such that at higher levels of sleep quality, the association between discrimination and depression symptoms was non-significant. Despite being cross-sectional, the results suggest that the risk of depression in PI youth involves post-adoption experiences that appear unrelated to the impacts of early deprivation on neurobiological processes associated with depression risk. It may be crucial to examine methods of improving sleep quality and socializing PI youth to cope with discrimination as protection against discrimination and microaggressions.

## Introduction

Youth adopted internationally from orphanage-like institutions have a set of experiences that afford the ability to study how adversity limited to early in life may increase the risk for depressive symptoms in youth and which factors post-adoption may confer additional risk and/or resilience from developing depressive symptoms. Youth adopted internationally from orphanage-like institutions experience marked improvements in their living conditions following adoption (van Ijzendoorn & Juffer, 2006). Despite these positive changes, many continue to face behavioral and emotional challenges, with a notable increase in depressive symptoms during adolescence and young adulthood (Colvert et al., 2008; Gunnar & van Dulmen, 2007; Sonuga-Barke et al., 2017). The underlying cause for this increase in depression is not entirely clear. The predominant hypothesis is that of a sleeper effect; that is, institutionally induced early life stress affects neurobiological systems associated depression risk that, with the occurrence of the structural brain changes of adolescence, markedly increase depressive symptoms (Golm et al., 2020; Whittle et al., 2014).

However, what is often left out of this argument is that post-adoption experiences for previously institutionalized (PI) youth may be involved and would be associated with depressive symptoms even after controlling for depression-associated neurobiological changes induced by early deprivation. The goal of this paper is to examine discrimination as a potential post-adoption depression risk factor for PI youth, after controlling for a measure of neurobiological functioning associated with depression risk that was induced by early deprivation. Peer rejection plays a powerful role in the emergence of depression in youth (Platt et al., 2013). PI youth differ from peers in ways that may lead to less acceptance. Specifically, they are adopted and some adoptees experience microaggressions for that reason alone (Baden, 2016; Garber & Grotevant, 2015). Indeed, parents report increased discrimination for PI youth adopted from Eastern Europe who are not from a racial or ethnic minority in the U.S. (Lee, 2010). Notably, however, many PI youth are transracially adopted. Thus, they are raised in White families often experiencing the paradox of not fitting in either with their racial-ethnic group or with their White family and peers (Anderson, 2015; Lee, 2003). Both adoption and racial and ethnic minority status may result in the experience of discrimination and otherness.

Employing a risk and resilience framework (Masten & Cicchetti, 2016), although PI youth encounter experiences that predisposes them to adverse developmental outcomes, they also have access to protective factors, both internal and external, that mitigate negative effects. Understanding sources of that resilience is another goal of this study. Sleep is critical to neuroprotection (Eugene & Masiak, 2015) and is a major stress regulator, providing a buffer against depression (da Estrela et al., 2021). Thus, the goal of this study was to examine the depressive symptoms of PI youth relative to a comparison sample of youth raised with their biological families (non-adopted, NA). After controlling for a measure of neurobiological vulnerability associated with early institutional care, we examined whether the experience of discrimination was associated with depressive symptoms and whether this association interacted with sleep quality in a way that suggested moderation.

## Depression risk

Early life stress in the form of neglect and abuse are well-established risk factors for adult depression (Cicchetti et al., 2010; Heim & Binder, 2012; Kim & Cicchetti, 2006). Rearing in an orphanage-like institution is a particularly severe form of early stress (van IJzendoorn et al., 2020). There is an extensive literature showing elevated risk of adjustment problems for PI children, especially when adoption follows longer periods of deprivation (van IJzendoorn et al., 2011). Nonetheless, elevated rates of depression have rarely been reported until adolescence. For example, among the Romanian children adopted into the United Kingdom, Rutter and colleagues reported problems with attention, quasi-autism, and attachment, but not emotional problems when children were pre-adolescent (Rutter et al., 2001). However, in early adolescence, PI youth adopted at 6 months or later did exhibit elevated emotional problems compared to adopted youth without early institutional experience (Colvert et al., 2008). By early adulthood, depression stood out as the significant psychological problem in the PI group (Sonuga-Barke et al., 2017). The increase in depressive symptoms with greater time-since-adoption was also noted in the analysis of a large sample of PI and comparison children adopted from many different countries (Gunnar & van Dulmen, 2007).

## Early life stress and autonomic balance

One source of increased depression risk may stem from impacts of early deprivation on stress-responsive systems (e.g., Adam et al., 2010). Autonomic balance, measured as the ratio of high to low frequency power in heart rate variability, is a well-studied risk factor altered in clinical depression (see meta-analysis, Koch et al., 2019). The autonomic nervous system is also altered by early life stress in the form of institutional care. We have noted alterations in sympathetic tone (pre-ejection period, PEP) under baseline conditions within week of removal from institutional care (Esposito et al., 2016) and years after removal and placement in supportive families (Gunnar et al., 2009). Altered baseline and response PEP have also been noted in the only randomized study of removal from institutional care (McLaughlin et al., 2015). In a previous study of PI adolescents, we noted altered autonomic balance with PI youth displaying a lower low frequency to high frequency ratio (LF/HF ratio; Reid et al., 2017). Thus, we covaried autonomic balance (LF/HF ratio) in the present study to partially reflect the aspect of depression risk induced by early institutional deprivation.

## Adoption status, discrimination, and depression

The international adoption literature highlights that adoption itself can empower children to nurture their potential for positive development through a permanent contextual change (Greene et al., 2008). Meta-analyses also show that it allows for massive catch-up in physical, cognitive, and social functioning (van IJzendoorn & Juffer, 2006). However, the stressors associated with adoption can contribute to later adjustment challenges. These stressors include post-adoption experiences, from ruminations about their birth families (Kim et al., 2020) to discrimination and microaggressions due to being an adopted individual and often also due to being Black, indigenous, people of color (BIPOC; Garber & Grotevant, 2015; Schires et al., 2020). These challenges coupled with typical adolescent developmental challenges can be a source of great stress for PI youth and set the stage for some of the adjustment problems they experience.

Discrimination, defined as unfair treatment based on group perceived membership (Slopen et al., 2016), not only induces general stress but also exerts a negative influence on adjustment. Racial discrimination, also a salient stressor during adolescents, elicits feelings of threat (Huynh & Gillen-O’Neel, 2016) and heightens vigilance or alertness (Majeno et al., 2018), potentially impairing emotional regulation in youth, leading to emotional dysregulation and sleep disturbances (Slopen & Williams, 2014). Notably, non-ethnic discrimination can similarly incite a sense of loneliness and social isolation (Majeno et al., 2023).

Discrimination is a well-studied correlate of increased depressive symptom and internalizing problems, although positive racial-ethnic identity development can serve as a partial buffer (Budescu et al., 2023; Yip et al., 2019). Unfortunately, many families that adopt international and transracially approach their multi-racial family group using a colorblind set of principles (i.e., we are all the same under our skin) and/or engage in limited ethnic socialization (e.g. culture camp, eating ethnic food) and do not engage in preparation for bias (Hu et al., 2017; Kim et al., 2013). Furthermore, being White and often financially privileged, they are ill-equipped through their own experiences to help their children cope with the various forms of discrimination and microaggression they will face (Lee, 2002).

## Sleep quality

Among the myriad stressors influencing psychological health among PI youth, the role of sleep quality emerges as a potential protective element. Adequate and restorative sleep is integral to the maintenance of cognitive functions, emotional regulation, and overall psychological resilience (Dahl & Lewin, 2002; Yip, 2015). Youth with high-quality sleep patterns are more likely to exhibit enhanced coping mechanisms in the face of stressors, acting as a buffer against the detrimental effects of external pressures. The current study delved into the potential moderating influence of the physiological factor of sleep. As a moderator, sleep can function as both a risk and protective factor. There is increasing evidence of high heritability of various aspects of sleep quality, including the susceptibility of sleep to disturbances by daily stressors in adults, children and adolescents (Barclay et al., 2011; Goel, 2017; Lewis & Gregory, 2021). Sleep disruptions are often characterized as shorter than average sleep times, increased activity during sleep, frequent night wakings, or variability in sleep schedules from night to night. Consequently, sleep quality is often negatively impacted by current and lifetime stressors (Majeno et al., 2023). In cases where youth manage to maintain higher sleep quality despite reporting similar stressors, we would expect that they would exhibit lower levels of depressive symptoms. Notably, sleep has been shown to moderate the relations between discrimination and outcomes in youth of color born in the United States, with high sleep quality serving to protect and poor sleep quality to increase the risk of negative effects of discrimination (El-Sheikh et al., 2022; Yip et al., 2023). Thus, sleep quality may contribute to the risk and resilience spectrum (Masten et al., 2021).

## Current study

In the following study, we examined depressive symptoms in adolescents and young adults who had been adopted internationally from institutional care when they were infants and very young children, most under 3 years of age. We compared them to individuals born and raised in families of comparable education and incomes. We obtained measures of autonomic balance (LF/HF ratio), discrimination, sleep problems, and depressive symptoms. We predicted that PI relative to NA youth would exhibit more depressive symptoms and would report more experiences of discrimination. After controlling for autonomic balance, which we expected to be altered in PI youth due to early life stress, we predicted that experience of discrimination would be associated with depressive symptoms, but that for youth who reported high sleep quality the association between discrimination and depressive symptoms would be lower or non-significant. We made these predictions recognizing that these were cross-sectional data, and thus causal interpretations would need to be made cautiously.

## Methods

### Participants

Participants were from Wave 1 of the longitudinal Early Life Stress and Cardiometabolic Health in Adolescence Study (CardioHealth Study). Ninety-three previously institutionalized (PI) youth who were adopted internationally from orphanage-like rearing institutions (58 females and 35 males; age range 12.02–21.39 years; Mean age = 16.32, SD = 2.42 years) and 95 youth raised in biological families (Non-adopted, NA; 49 females and 46 males; age range 12.11–21.82 years; Mean age = 15.24, SD = 2.35 years). See demographics Table 1. All participants, or their parents in the case of college students, currently resided within driving distance of the University of Minnesota Clinical and Translational Science Institute (CTSI). The PI youth had entered their adoptive parents fulltime care between 2 and 60 months of age, with a mean of 16.01 months, SD = 11.98 and median of 12 months. The average percentage of their lives lived in institutional care prior to adoption was 91%. At the time of assessment, the PI youth had been in their adoptive families for an average of 14.98 years (*SD* = 2.5 yrs).

The University of Minnesota Institutional Review Board approved all procedures. All minor participants assented and their parents provided informed consent, while participants who reached the age of majority provided consent.

### Protocols & measures

Participants were drawn from two sources. PI youth were drawn from a registry of families who adopted internationally and who were interested in being contacted about research opportunities maintained since 2001 by our research group. NA youth were contacted through the department’s Participant Pool. This pool consists of families who indicated that they were interested in being contacted for research opportunities following a mailing based on county birth records obtained soon after the child’s birth or after signing up at the University’s booth at the Minnesota State Fair. This registry tends to contain names of children in families of generally well-educated and higher income parents, which makes them comparable to families who adopt internationally.

Participants who met inclusion and exclusion criteria were invited to participate. Inclusion criterion for the NA youth was that they were born into and raised by their biological family. Inclusion criteria for the PI youth were that they had been adopted from orphanage-like institutions after having spent 50% or more of their lives in institutional care. Exclusion criteria for both groups were: known premature birth (less than 37 weeks of the gestational age), congenital and/or chromosomal disorders (e.g., cerebral palsy, FAS, intellectual disability disorder, Turner Syndrome, Down Syndrome, Fragile X), history of serious medical illness (e.g., cancer, organ transplant), taking systemic glucocorticoids, and taking beta-adrenergic medications unless they could forego beta-adrenergic medications for at least 24 hours prior to testing.

Wave 1 data were collected between March of 2021 and February of 2023. Wave 1 involved 2 sessions, with session 1 conducted virtually and session 2 conducted at the CTSI in the university’s health science center. During session 1, youth participants and their parents completed online questionnaires via the web-based software REDCap (Harris et al., 2009). Youth came to session 2 after fasting for at least 8 hours. During session 2 participants underwent a comprehensive cardiometabolic health check. A small subset of the measures collected at Wave 1 were included in the present analysis.

### Measures

#### Demographics

Demographic information provided by the participants included birthdate (recalculated using the testing date as participant’s age), parent education and income, race and ethnicity, and sex assigned at birth. For the PI youth, demographics also included age when the child came into the parent’s fulltime care, months in an institution/orphanage, and country of birth recoded into birth region (Latin America and Caribbean, Russia and Eastern Europe, African Continent, and Asia). The parent who was present at the Wave 1 session 1 visit completed the parent questionnaire. In most cases (91%) this was the mother. See Table 1.

#### Depressive symptoms

Parents completed the MacArthur Health and Behavior Questionnaire (HPQ-P 2.11, Essex et al., 2002) from which we obtained scores for depressive symptoms. The depressive symptoms scale consists of 18 items with responses 0–2. The alpha for the scale in this sample was .90. The parent-report measure of depressive symptoms, rather than the youth report version was used to avoid the problem of the same person reporting on both the independent and dependent variables in the regression. There were 4 participants (all female, one PI) who were missing this parent-report measure. These were the only 4 missing data points in the whole analysis. Because depressive symptoms were the dependent measure and only 2% were missing, we decided not to impute the missing values. The depressive symptom scale was positively skewed and was thus log10 transformed after addition of a constant to adjust for 0 scores.

#### Low frequency/high frequency ratio

The LF/HF ratio measure was obtained during the clinic visit at session 2. Participants arrived fasting (8+ hrs) and the measure of autonomic balance was obtained about 30-45 minutes into the session and prior to the blood draw for the metabolic panel (not reported here). LF/HF Ratio as an index of autonomic balance was assessed from measures of heart rate variability derived from SphygmoCor MM3 version 8.0 software (AtCor Medical, Sydney, Australia) used to assess carotid radial pulse wave velocity (not reported here). Using a 3-lead ECG in a modified lead II configuration, heart rate was recorded continuously for 5 minutes. ECG recordings were then reviewed and analyzed for time and frequency domains. Segments demonstrating arrhythmias were excluded from analysis. Spectral analysis calculated frequency domains and yielded our measure of LF/HF ratio. Frequencies of 0.04–0.15 Hz and 0.15–0.40 Hz were defined as LF and HF, respectively (Ryder et al., 2016).

### Discrimination score

This score was the combination of two discrimination scales, standardized and averaged. Both scales were completed by the youth participants. To explicitly address discrimination based on race/ethnicity, we used the 3-item racial denigration subscale (alpha .88) of the *Brief Discrimination scale*

(BDS; Armenta et al., 2013). This scale used a 4-point Likert-response format (never to 5 times or more) and examined experiences in the last year, including: Rejected by others because of your race/ethnicity, denied opportunities because of your race/ethnicity, and treated unfairly or rudely by strangers because of your race/ethnicity. The *Everyday Discrimination Scale* (EDS; Williams et al., 1997) measures chronic and routine unfair treatment in everyday life, not necessarily related to race or ethnicity. Respondents report unfair treatment in their day-to-day life on a 6-point Likert-type format. Responses ranged from 1 (never) to 6 (almost every day). The scale included nine items (alpha .88): You are treated with less courtesy than other people are, you are treated with less respect than other people are, you receive poorer service than other people at restaurants or stores, people act as if they think you are not smart, people act as if they are afraid of you, people act as if they think you are dishonest, people act as if they’re better than you are, you are called names or insulted, and you are threatened or harassed. The EDS also asks respondents to indicate why they think they were discriminated against, offering multiple options from race to weight to age, and so on. Being adopted, however, is not one of the options, though could be reported under “other”. These were explored to better understand the perceived reasons for discrimination.

### Poor sleep quality

Sleep quality, as reflected largely in daytime sleepiness, was indexed using scales from the Cleveland Adolescent Sleepiness Questionnaire (CASQ; Spilsbury et al., 2007) and the Brown School Sleep Habits Scale (BSSHS; Wolfson & Carskadon, 1998).Youth participants self-reported on frequency of sleep-related problems they experienced during the day, for example: whether they “arrived late to class because of oversleeping,” “stayed up all night,” “feel tired, dragged out, or sleepy during the day,” “felt (dis)satisfied with sleep,” “had a (poor) night’s sleep,” “struggle to stay awake in class.” The CASQ yields 2 scales: *daytime sleepiness* (11 items, alpha = .84) and *daytime alertness* (5 items, alpha = .88). The BSSHS yields 3 scales of daytime functioning: *sleepiness* (10 items, alpha = .75), *sleep-wake behavior problems* (10 items, alpha = .81), and *sleep quality* (2 items, alpha = .81). Scales were reversed so all were scored in the direction of poor sleep quality and subjected to a principal component analysis that yielded only one factor that accounted for 63% of the variance. A factor score was produced and labeled Poor Sleep Quality.

### Data analysis plan

Demographic data were first analyzed to determine the complementarity of the two groups. Next group by sex analyses were computed on the key variables. We also examined the top 3 reasons given for discrimination on the EDS for each group. Intercorrelations were then examined to determine whether key independent variables could be included in the same regression analysis. Finally, a hierarchical regression with *R*^2^ change statistics was computed with depressive symptoms as the dependent measure. Covariates of sex and age were entered in the first model, then Group, then LF/HF ratio, then Discrimination, then Sleep Problems, then the centered interaction of Discrimination and Sleep Problems. Analyses were conducted using IBM SPSS Statistics, Version 27.

## Results

### Demographics

As shown in Table 1, the two groups were comparable in youth sex assigned at birth, family income and parent education. Median family income after taxes was high, placing these families roughly in the upper 20% of the population. Parent education was quite high, with 84% of the NA and 95% of the PI parents having a four-year college degree or more. This compares to the state average of 38% of adults. Most youth arrived with a parent, nearly always a mother; more PI participants arrived unaccompanied (18+yrs). Most of the parents of both groups were White and Non-Hispanic. In contrast, nearly all (84%) of the PI youth were BIPOC living with White parents. Almost half of the PI youth were from Asia, including Nepal and India in addition to China and Vietnam. About a quarter were from Latin America and the Caribbean. Smaller numbers were from Russian and countries in Eastern Europe and Africa.

### Depression

As shown in Table 2, PI youth were described as exhibiting more depressive symptoms than NA youth. The sex difference was not significant, although there was a marginally significant sex by group interaction. Examination of the means suggests that this reflected the fact that the PI boys, unlike the NA boys, were described as exhibiting as many depressive symptoms as the girls. The Cohen’s *d* (Cohen, 1992) effect size for the group difference was .36.

### LF/HF ratio

Also in Table 2, the results for the measure of autonomic balance indicated a significant group difference. The PI youth exhibited a lower ratio than the NA youth, with a Cohen’s *d* of .38. Again, the sex difference was not significant.

### Discrimination

PI participants reported more intense experiences and more frequent experiences of discrimination than did the NA participants (see Table 2). The Cohen’s *d* effect size was .32. Again, the sex difference was not significant. An examination of the reasons given for discrimination in response to the EDS showed that the top 3 reasons for NA youth were gender, age, and physical appearance. For the PI youth it was race/ethnicity/skin color, gender, and age.

### Poor sleep quality

The group difference in sleep quality was not significant (see Table 2). There was a large sex difference, with females describing poorer sleep than males.

### Intercorrelations

Intercorrelation of the independent variables are shown in Table 3. LF/HF ratio was not correlated with either discrimination or poor sleep quality. Discrimination and poor sleep quality were positively correlated but the magnitude of the association (they shared only 12% of their variance in common) did not preclude entering both into the same regression analysis. All three measures (discrimination, sleep quality and LF/HF ratio) were significantly correlated with depressive symptoms in the bivariate correlations, with the first two measures being positively and the last being negatively correlated.

### Regression analysis

**Figure 1.**
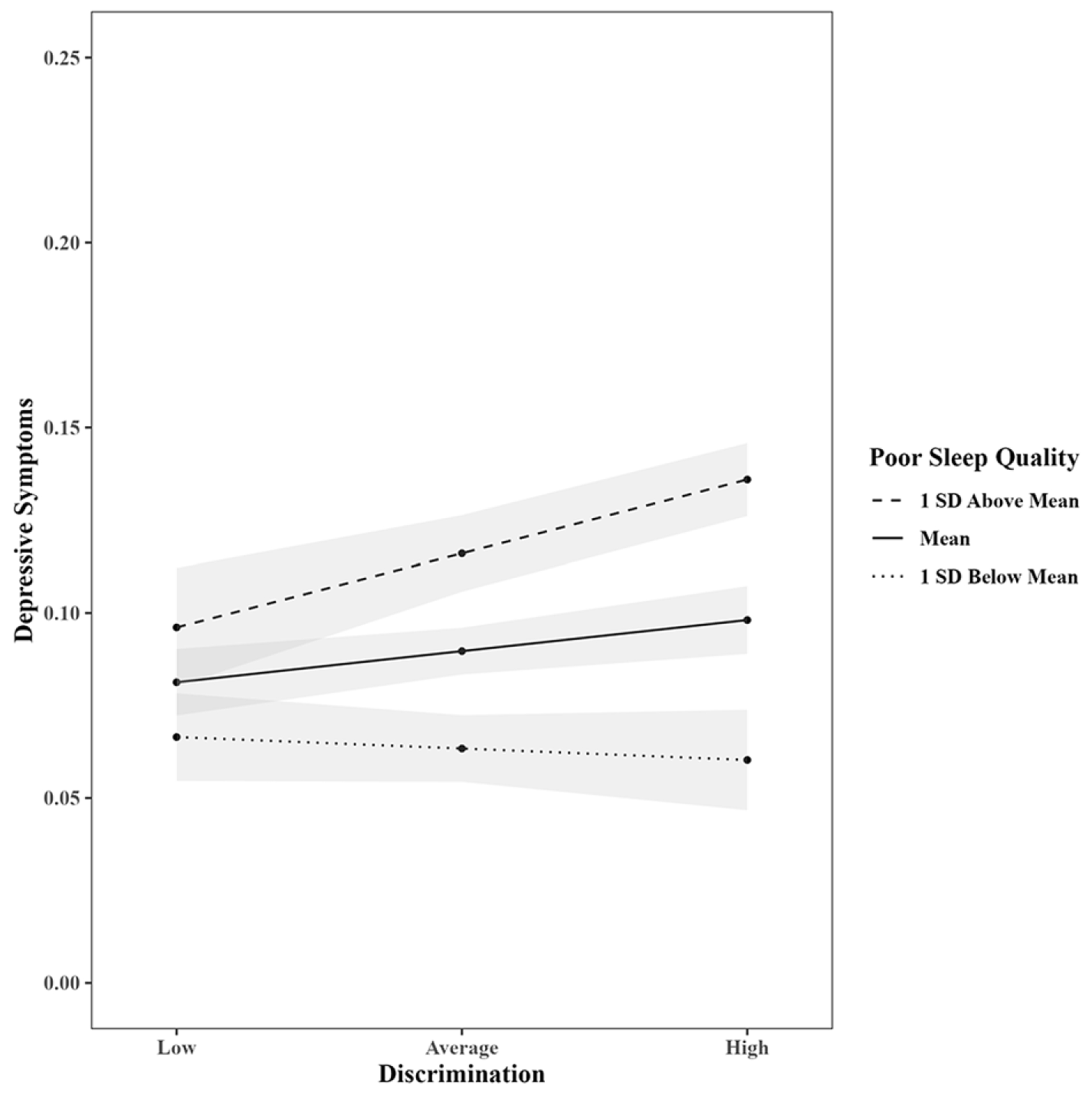
Plot interaction between discrimination and poor sleep quality predicting depressive symptoms. The plot depicts the significant interaction between discrimination and sleep predicting depressive symptoms. The *X* axis represents the frequency and intensity of youth reported discriminatory experiences. The *Y* axis represents the continuous range of values for parent reported depressive symptoms. The dashed lines are plotted at 1 SD above and below the mean. Poor sleep quality is measured via youth reported measures. As shown, sleep quality significantly moderates the association between experiences of discrimination and depressive symptoms as those youth reporting better (less poor) sleep having little to no increases in depressive symptoms with increased experiences of discrimination.

The results are shown in Table 4. After entering the two covariates of sex and age, adoption status, dummy scored as 0 (NA) 1 (PI) was entered and significantly increased the prediction of depression. Adding LF/HF ratio did not significantly improve the model however it reduced PI status to non-significance. In each of the next two steps, adding discrimination and then poor sleep quality improved the variance explained. In the last step, adding the interaction of discrimination and poor sleep quality also significantly increased the variance explained. The final model accounted for 18% of the variance (using adjusted *R*^2^). Figure 1 shows the interaction of discrimination and sleep in relation to depressive symptoms, and the upper and lower 1SD lines. The pattern clearly indicates that at higher levels of experienced discrimination, youth who were able to maintain high sleep quality exhibited a near zero association between discrimination and depressive symptoms. The reverse was also true, that even at lower levels of perceived discrimination, poor sleep quality was associated with a higher association between discrimination and depressive symptoms.

## Discussion

We found that PI youth exhibited more depressive symptoms, but entering our measure of autonomic balance reduced that association to non-significance, suggesting that it served as a reasonable control for the aspect of increased depression risk associated with neurophysiological changes induced by institutional care. In both groups of youth, discrimination experiences were associated with elevated depressive symptoms; nonetheless, PI youth reported more of these experiences than NA youth. Sleep quality did not differ for PI and NA youth; however, as predicted, youth reporting more discrimination who also reported better sleep quality did not exhibited elevated depressive symptoms, and vice versa. Thus, sleep appeared to serve as a moderating factor, protective when good and a risk factor when poor.

Although this was a convenience sample, the PI youth in this report were typical of those adopted internationally into the U.S. As with other PI youth, they were adopted at an early age, typically before age 3. They had spent nearly all of their preadoption lives in institutional care that would deprive them of the human interaction necessary for healthy neurobiological, development and also expose them to high levels of pathogens both factors being chronic stressors (van IJzendoorn et al., 2011). When we assessed the PI youth in the present study, they had been in their adoptive families for an average of nearly 15 years. Their adoptive parents were highly educated and well-resourced. In this respect, the adoptive parents were comparable to the parents of the NA youth. Where they were not comparable was in their racial and ethnic similarity to their children. Among the NA families, all of the children and parents shared their race and ethnicity. Among the PI families, nearly all of the parents were White, while 84% of the PI youth were from what is considered a racial or ethnic minority in the U.S. While adoption meant removal from deprivation and provision of a family, at the same time it created challenges of otherness (Lee, 2002). Thus, PI youth experienced at least two potential sources of depression risk, one source induced by their early experiences of deprivation and neglect and the physiological and neurocognitive sequelae of that early deprivation, and the other arising post-adoption from the challenges of otherness: i.e., being adopted and becoming a member of a racial or ethnic minority group in the U.S.

### PI status and LF/HF ratio and depressive symptoms

We previously conducted a study of cardiometabolic health in PI youth who were height stunted at adoption and found that compared to youth raised in their birth families the PI youth exhibited a reduced LF/HF ratio (Reid et al., 2017). The present study replicated those results without an inclusion criteria of linear growth faltering. Furthermore, we obtained a negative bivariate correlation between this measure of autonomic balance and depression. In previous work using pre-ejection period (PEP), a more direct measure of sympathetic tone, we and other have found higher sympathetic tone at baseline for PI youth, but a blunted sympathetic response to psychosocial stressors (Esposito et al., 2016; Gunnar et al., 2009, McLaughlin et al., 2015). While the low frequency value in the LF/HF ratio is often interpreted as reflecting sympathetic tone, this is not accurate (Billman, 2013). Indeed, among adolescents, PEP has been shown to be either uncorrelated with or positively correlated with the low frequency measures, which would indicate that higher sympathetic tone (PEP) was associated with lower low frequency heart rate variability for adolescents consistent with our previous findings (Goedhart et al., 2008). Whatever the association of the autonomic balance measure of heart rate variability is with more direct measures of sympathetic tone, it seems clear that for PI adolescence there is a shift towards a lower ratio. Notably, a lower LF/HF ratio has been shown to be associated with reduced glucose metabolism and thus a reduced use of metabolic resources (Plaza-Florido et al., 2002). This may well be adaptive under conditions of deprivation. Indeed, many of the physiological differences between PI and NA children as well as those observed while young children are in institutional care can be interpreted as shifts to reduce energy expenditure. For example, children in institutions and PI youth at adoption exhibit stunted linear growth and blunted cortisol responses to stressors (see review, Gunnar & Reid, 2019). Both linear growth and increases in cortisol (a glucocorticoid) burn energy. Thus, this shift in LF/HF ratio at baseline may be another reflection of calibration of energy-demanding systems to a lower set-point under conditions of deprivation. As expected, this measure of autonomic balance was associated with depressive symptoms and, when entered into the model, reduced the association of PI status with depressive symptoms.

### Group differences in depression symptoms and discrimination

In examining the data on depressive symptoms, it is important to contextualize the study in relation to the COVID-19 pandemic which appeared to increase affective disorders in adolescents, especially those of higher income such as the youth in this study (Madigan et al., 2023). Overall, as expected, the PI youth were described as exhibiting more depressive symptoms than NA youth with an effect size that was between small and medium (.36, Cohen, 1992). There are no validated cut-points for the HBQ Parent Report 2.1 and we did not conduct clinical assessments, thus we do not know how many of the PI and NA youth met clinical criteria for depression.

PI youth reported more intense and frequent experiences of discrimination than NA youth, with their top three reasons for experiencing discrimination between related to racial and ethnic discrimination compared to age and gender for NA youth. Racial, cultural, and other phenotypic distinctions between adoptive parents and children add complexity to their adaptive trajectory. Consequently, navigating through intrusive and offensive comments or other discriminatory experiences becomes particularly challenging due to factors such as adoption status or being part of a racial minority (Lee, 2010). Notably, experiences of any type of discrimination were associated with increased depressive symptoms for both PI and NA youth. Importantly, because data on depressive symptoms and perceived discrimination were obtained concurrently, we cannot rule out the possibility that youth who are experiencing more depressive symptoms report more experiences of discrimination. That is, we need to be cautious about the direction of the causal arrow. These data were from wave 1 of this study, with data in wave 2 (2 years later) in the process of being collected. Thus, we will be able to determine whether perceived discrimination in wave 1 predicts the change in depressive symptoms from wave 1 to 2. Regardless of the causal relations, youth experiencing discrimination, especially for characteristics like race and ethnicity, need support in developing coping mechanisms to navigate both structural and interpersonal forms of discrimination. Racial and ethnic socialization for PI youth of color could be an avenue where strategies to cope with and withstand the discrimination they experience in the U.S. (Budescu et al., 2023; Huet al., 2017; Kim 2013), in addition to learning about customs and traditions of their birth country.

### Sleep as a protective factor

Protecting sleep quality would be another way of reducing the negative impact of experiencing discrimination and microaggressions. Sleep quality tends to decline during adolescence due to numerous social factors (Paavonen et al., 2010). Additionally, it has been argued that early life adversity should be associated with poorer sleep quality (Fuligni et al., 2021). However, there is little evidence that PI youth adopted early from institutional care suffer from more sleep problems as adolescents and young adults. Indeed, in the present study they reported the same sleep quality as the NA youth. Sleep quality was, however, negatively correlated with discrimination. This is consistent with many arguments about discrimination being a stressor that impairs sleep and increases the risk of mental health problems (e.g., Slopen et al., 2016). However, as noted earlier in the paper, there is a high heritability to most parameters of sleep quality, including the capacity to maintain better sleep even under conditions of stress (Barclay et al., 2011; Goel, 2017; Lewis & Gregory, 2021). Furthermore, sleep is critical to regulation of stress and serves as a buffer reducing the effects of stress on depression (El-Sheikh et al., 2022). In the present study, consistent with hypothesis, the association of discrimination with depressive symptoms was statistically moderated by the quality of sleep. Specifically, individuals who reported experiencing discrimination but also reported having higher quality sleep demonstrated lower levels of depressive symptoms compared to those who experienced discrimination and had lower sleep quality. This finding plus others that are similar but that allow more causal inference, suggests that working to support good sleep habits may be especially important in buffering youth, and particularly PI youth of color from the stressors they will experience as adolescents and young adults. This interaction implies that maintaining good sleep quality may act as a protective factor, mitigating the negative effects of discrimination on mental health; while poor sleep quality can have the opposite effect. Thus, preventive interventions could focus on promoting and improving sleep quality as a targeted strategy to mitigate the negative impact of discrimination on mental health.

### Limitations & future directions

Though this study presents evidence that experiences outside of early deprivation need to be considered in the study of PI youth, there are limitations to note. Again, this study was conducted in the two years following the development of vaccines for the COVID-19 virus. Our earliest participant was tested in April of 2021. Thus, depressive symptoms may have been elevated for both PI and NA youth. Our measure of sleep was by self-report, which often is not correlated with objective measures of sleep (Cudney et al., 2022). It will be important to include both subjective and objective measures of sleep in future studies of discrimination, depressive symptoms, and sleep quality in PI youth and in studies of youth of color. As we have repeatedly noted, this was a cross-sectional analysis of our first wave of data and thus we cannot be confident of the causal relations among our variables. The present results, however, will serve as a guide to our analyses of the wave 1 to wave 2 data where we can obtain a better assessment of the causal relations. We also used parent report of youth depressive symptoms rather than youth report in order to reduce the problem of correlation across self-report measures. However, this may have reduced the reliability of the depressive symptoms measure as parents may not be aware of how their youth is feeling internally. Despite these limitations, there were several obvious strengths. First, the sample size is relatively large for a study of PI youth. In addition, the youth were from many different countries, thus increasing the generalizability of the findings. Moreover, the comparison youth were raised in homes of similar economic and educational resources.

## Conclusion

In summary, the relationship between discrimination, autonomic balance, sleep quality, and depression among PI and NA youth reveals a complex interplay of factors. Discrimination, stemming from sources like daily hassles, racial disparities, and the unique challenges faced by transracially adopted youth, shows a significant correlation with mental well-being, even after adjusting for autonomic balance, which is often linked to depression and strongly influenced by early deprivation. Clearly, efforts to improve mental health outcomes for youth of color who have experienced early life stress need to focus on reducing structural and interpersonal discrimination. In the meantime, programs that support better sleep quality among youth would be expected to support resilience in the face of discrimination. The present findings, taken together with those of Cicchetti et al., 1993, Cicchetti, 2013, Cicchetti & Banny, 2014, for example, advance developmental psychopathology research by shedding light on the conditions necessary for normal development and healthy adaptation following early life stress. Importantly, these results point to the importance of considering post-adoption factors, including those created by the society into which the PI youth has been adopted to understand their mental health risk.

## Figures and Tables

**Table 1.** Study demographics

	Post-Institutionalized	Non-Adopted	Significance
% Female	62%	52%	ns
Median Family Income	100,000–150,000	100,000–150,000	ns
% Parents with 4-yr College Degree	99%	87%	ns
% Parent 1 White	95%	84%	*p* =.02
% Parent 2 White	72%	81%	ns
% BIPOC Youth	8%3	16%	*p* < .001
Birth Region			
USA		100%	
Latin America and Caribbean	24.2%		
Russian and Eastern Europe	15.4%		
African Continent	13.2%		
Asia^1^	47.3%		

1 Includes Nepal and India.

**Table 2.** Univariate analyses of variables in the regression with means (SD) and *F* statistics

Variable	PI		NA		*F* (1,184) Group	*F* (1,184) Sex	*F* (1, 184) Sex X Group
	Female	Male	Female	Male			
Depression	0.11 (0.01)	0.11 (0.02)	0.10 (0.01)	0.06 (0.01)	5.33*	1.34	3.16
LF/HF Ratio	−0.37(0.05)	−0.37(0.06)	−0.26(0.05)	−0.19(0.06)	7.40*	0.31	0.31
Discrimination	0.15(0.13)	0.18(0.17)	−0.08(0.14)	−0.24(0.15)	4.79*	0.20	0.48
Sleep Quality	0.31(0.13)	−0.10(0.16)	0.18(0.14)	−0.50(0.14)	3.46	14.74**	0.94

Note.

* =p < .05

** =p < .01.

**Table 3.** Correlations for key study variables

Variable	1	2	3	4
1. Depression	–			
2. LF/HF Ratio	−.15*	–		
3. Discrimination	.23*	.01	–	
4. Sleep Quality	.38**	−.06	.37**	–

Note.

* =p < .05,

** =p < .01.

**Table 4.** Linear regression with unstandardized coefficients predicting depression

Variables	Models					6
	1	2	3	4	5	
Sex	−.02(.01)	−.02(.01)	−.02(.01)	−.01(.01)	<.001(.01)	<.001(.01)
Age	−<.001(.00)	−<.001(.00)	−<.001(.00)	−<.001(.00)	−.01*(.00)	−<.001(.00)
Adoption Status		.03*(.01)	.03*(.01)	.02(.01)	.02(.01)	.02(.01)
LF/HF Ratio			−.03(.02)	−.03(.02)	−.02(.02)	−.03(.02)
Discrimination				.09*(.03)	.05(.03)	.04(.03)
					.03**(.01)	
Sleep Quality						.03**(.01)
Interaction						.01*(.01)
Constant	0.12(0.05)	0.13(0.05)	0.12 (0.05)	0.14 (0.04)	0.16(0.04)	0.13(0.04)
*R* ^2^	0.01	0.04	0.05	0.10	0.19	0.21
*F R*^2^ *Change*	1.17	5.21*	2.52	8.64**	20.71**	3.92*
*F Model*	1.17	2.54	2.55*	3.85*	7.02**	6.67**

Note.

** *p* < .01;

* *p* < .05;

Values in parentheses represent standard errors.
